# Representation and methods of normalisation: Narratives of disability within a South African tertiary institution

**DOI:** 10.4102/ajod.v9i0.629

**Published:** 2020-07-23

**Authors:** Taegan Devar, Shaida Bobat, Shanya Reuben

**Affiliations:** 1 People Smart Consulting, Durban, South Africa; 2 School of Applied Human Sciences, Discipline of Psychology, University of KwaZulu-Natal, Durban, South Africa

**Keywords:** SWD, normalisation, narratives, social constructionism, higher education institutions

## Abstract

**Background:**

The manner in which disability is understood influences how individuals within a society, its institutions, policies and structures are able to accommodate and support people with disabilities (PWD) (Kaplan 2000). Understanding how students with disabilities (SWD) within a higher education context perceive and experience disability as well as how key players, namely, lecturers and disability unit (DU) staff, who influence that experience, is important in further shaping policy and providing a truly inclusive environment for all within HEIs.

**Objectives:**

The study aimed to examine the narratives of disability among SWD, lecturers and the DU within a tertiary institution, with a view to better understand their experiences and required initiatives to address the challenges of disability within a higher tertiary institution.

**Method:**

The study drew on three theoretical frameworks: social constructionism, feminist disability theory and the Foucauldian perspective. Data for the study were collected through in-depth semi-structured interviews with 12 SWD, seven members of staff from the institution’s DU and five lecturers from within the School of Applied Human Sciences. Data were analysed using thematic analysis.

**Results:**

The findings suggested that in spite of both facilitating and positive representations of disability, the dominant representation of disability was perceived as challenging and as a result, disempowering. Students with disabilities were found to adapt, and consequently modify their behaviour by disassociating from their disability in order to fit in.

**Conclusion:**

The study highlights the need for creating spaces and engagement within an HEI context that both challenge negative discourses of disability, and at the same time, promote positive representations of disability.

## Introduction

### Social and scientific value

For most students, studying at the tertiary level is an empowering experience; however, for many students with disabilities (SWD), this empowering experience of higher education is often difficult to achieve (Fuller, Bradley & Healy 2010). In 2011, an estimated 7.5% of South Africans reportedly were living with a disability that prevented them from full participation in life activities (including equal access to higher education). Although no research has been conducted on the prevalence of SWD within higher education institutions (HEIs) in South Africa, it is estimated that in 2011, SWD made up less than 1% of the student population of many HEIs in the country (Statistics South Africa 2014). This is alarming, especially given the provisions of the South African education system to support SWD that are founded on a human rights framework and promotes inclusivity (Matshedisho 2007). Mutanga (2017) argued that a contributing factor to the low participation in South African HEIs is the limited support provided by institutions, as disability matters are not prioritised (Ohajunwa et al. 2014; Tugli et al. 2013). Furthermore, commitment and support at the government level is a challenge (Matshedisho 2007; Matunga 2017). Consequently, access to HEIs does not guarantee that SWD will be able to access education and be successful when they enter HEIs (Mutanga 2017). Barriers as a consequence of South Africa’s apartheid era have influenced the manner in which HEIs are structured and function, as well the dominant beliefs and attitudes that inform practices within HEIs (Howell 2006, Matunga 2017). These barriers are problematic, because postsecondary experiences are vital in shaping students’ beliefs, identity and self-concept (Hutcheon & Wolbring 2012; Kraus 2008), and also because they impact on students’ health and access to future opportunities (Jung 2001). Furthermore, the experience of tertiary education provides a means for people with disabilities (PWD) to participate in knowledge production and policy development that describes their own perspectives (Jung 2001).

According to the Foundations of Tertiary Institutions of the Northern Metropolis (FOTIM) report on disability in higher education (Healey, Pretorius & Bell 2011), there is no single definition of disability that exists within the South African tertiary sector (Healey et al. 2011). Rather, different HEIs have their own way of classifying disability and SWDs (Healey et al. 2011). The model adopted by HEIs has a significant impact on the kinds of services provided and the manner in which they are provided (2011). The definitions utilised by HEIs suggest that a conceptualisation of disability within a medical model framework is still predominant; however, there is a shift towards an acknowledgement of external factors in ensuring inclusivity (Healey et al. 2011; Mutanga 2017). Foundations of Tertiary Institutions of the Northern Metropolis’s study further highlighted this, explaining that there is still a predominant focus on impairment and an individual having to fit into and adjust to the environment (DMS 2011). It is argued that a common definition of disability needs to be formed for South African HEIs that express the fluid nature of disability as a concept as well as acknowledging the functional impairment and barrier elements against which an individual can be assessed (Healey et al. 2011).

The World Report on disability (World Health Organization [WHO] 2011) provided a balanced approach to disability and acknowledged different aspects of disability (WHO 2011). The International Classification of Functioning, Disability and Health (ICF) understands disability and functioning as a dynamic interaction between contextual factors and health conditions known as the bio-psycho-social model of disability (WHO 2011). Disability within this framework is understood as a broad term for ‘impairments, activity limitations and participation restrictions’ (WHO 2011:7), referring to the negative influences of interaction between the individuals who have a particular health condition and personal and contextual factors (WHO 2011). Wheeler (2011) described how a conceptualisation of disability that takes into account the complex interaction between the individual and society and accounts for the complex variability in social, perceptual and behavioural characteristics that occur in PWD creates an understanding of disability not as a deficit but rather as a perceptual difference.

Shakespeare (2014) emphasised the importance of this point, explaining that there are several reasons as to why biological and social factors are interdependent. Impairment is a necessary condition in understanding the challenges facing those with disability (Shakespeare 2014). Consequently, it has to be acknowledged as part of the definition of disability. Secondly, much impairment is often caused by social conditions (Shakespeare 2014).

Furthermore, these impairments are often exacerbated by social conditions or processes (Shakespeare 2014). Therefore, a definition of disability that takes into account the dynamic relationship between these factors such as the WHO’s bio-psycho-social framework of disability enables a greater understanding of people with disabilities’ experiences and the manner in which they navigate their social context.

Disability studies in the context of higher education are readily available ranging from academic performance, unequal opportunity, support services, identity and, among other things, barrier and enabler factors (Mosia & Phasha 2017; Mutanga 2017, 2018). It is argued that a common definition of disability needs to be formed for South African HEIs that expresses the fluid nature of disability as a concept as well as acknowledging the functional, impairment and barrier elements against which an individual can be assessed (Healey et al. 2011; Mutanga 2017).

Historically, PWD in South Africa have been discriminated against, marginalised and have been prevented from exercising fundamental political, economic, social, cultural and development rights (South African Human Rights Committee 2002). This was a result of the predominant view of PWD within a medical framework, which viewed PWD as sick and in need of care rather than as equal citizens with responsibilities and rights (Howell, Chalklen & Alberts 2006). Inequalities in the schooling system exacerbated the challenge, as learners were separated based on both racial lines as well as on the basis of who were ‘normal’ and who had ‘special needs’. In response, two schooling systems emerged: (1) a dominant mainstream system for ‘normal’ learners and (2) a special education system for those with special needs. This secondary system had a limited number of resources and classes within mainstream schools, especially for black learners with disabilities, resulting in high levels of exclusion from the education system (Howell 2006; Swart & Pettipher 2011). This had a direct knock-on effect on the number of SWD who had access to higher education.

In spite of the enabling legislation, policies and guidelines to support PWD in HEIs, the management of disability continues to be fragmented, and the commitment of HEIs towards PWD, including the allocation of resources in supporting PWD, continues to be varied (Strategic Policy Framework on Disability for the Post-School Education and Training System 2018). This includes the manner in which HEIs are structured, how they function, the dominant attitudes that influence practices and the role that higher education plays in society (Healey et al. 2011; Strategic Policy Framework on Disability for the Post-School Education and Training System 2018). Students with disabilities face resource constraints, minimal student or teacher interaction and poor awareness of disability issues within the tertiary community (Healey et al. 2011; Mosia & Phasha 2017; Naidoo 2010). In addition, SWD are often placed in specific fields of study (Healey et al. 2011), subjected to the continued use or perspective of the medical model (Riddel 1998) and lack flexibility regarding curricula, inclusive learning and teaching methodologies (Healey et al. 2011; Mosia & Phasha 2017; Naidoo 2010).

Initiatives and structures to support SWDs in South African HEIs differ significantly across institutions in relation to the work that is carried out and the services that are offered (Healey et al. 2011; Shevlin et al. 2004; Strategic Policy Framework on Disability for the Post-School Education and Training System 2018). Disability units (DUs) within HEIs are often the first access point for students to receive support (Naidoo 2010). Services include awareness raising, policy development, the provision of assistive devices and equipment, assisting where access issues arise, auditing physical accessibility, provision of a dedicated computer room, providing personal and academic support, providing specialist services (such as a sign language interpreter), providing assistance with governmental bursary and grant applications, dedicating extra time for tests and exams, and providing support such as negotiating when conflicts arise (Healey et al. 2011; Matshedisho 2010; Naidoo 2010; Pillay et al. 2013). Furthermore, the willingness and attitudes of academic staff toward providing support to SWD influence the progress of these students in HEIs (Fuller et al. 2004; Mutanga 2017). Mutanga (2017) highlighted how academic staff often have different understandings and experiences of disability. This varies across faculties and staff members, and one of the many reasons for this is the absence of embedded HEI disability policy and practices (Mutanga 2017). Furthermore, support for SWD is reliant on DUs and individual academic staff members highlighting the need to understand the attitudes and views of academic staff with regard to SWD (Mutanga 2017).

There is a need for research that focusses on the lived experience of disability and those living with disability (Hurst 1996; Matunga 2017), and as Wheeler (2011:849) appropriately describes it: ‘the best person to say what support they need to access society is the individual who is experiencing it’. Therefore, understanding how SWD within a higher education context perceive and experience disability as well as how key players who influence that experience, namely, lecturers and the institutions’ disability support unit, perceive and experience disability is important in providing a truly inclusive environment for all within a tertiary institution.

Furthermore, there are few studies that are contextualised within the unique South African context in spite of the implications that this type of research bears for practice-level planning and policy-making in the area of disability.

### Conceptual frameworks

The study draws from three theoretical frameworks, namely, social constructionism, feminist disability theory and a Foucauldian perspective in order to understand how SWD, lecturers and the DU staff navigate disability within a tertiary context.

Within a *social constructionist framework*, disability is understood as an outcome of specific cultural conditions (Priestley et al. 2010; Siebers, 2001). Through language, understandings of disability are constantly being constructed and perpetuated in society (Burr 1995; Durrheim 1997). An individual’s sense of self is perpetuated through stories that are narrated about the self and reality. People with disability structure their narratives in relation to dominant cultural narratives that shape and become the context of their lived experience (Andrew 2004).

Dominant narratives about disability provide the opportunity of identifying what is understood as the normative experience in a particular context (Andrew 2004). When people’s experiences do not fit in with the dominant and the familiar, individuals question the foundations of these storylines and challenge them (Andrew 2004). Consequently, counter narratives are constructed and although they may understand their narratives as marginalised voices, they do not see them as unique (Andrew 2004). Therefore, marginal groups in society, such as the people with disabilities, are able to have their voices heard, highlighting perspectives and understandings that have been devalued, suppressed and abnormalised] (Delgado 1995).

Furthermore, personal stories around disability provide individuals with a chance to take on conceptions and ways of being that may be more facilitating (Andrew 2004) and may be more in line with their personal understandings. Highlighting this process allows individuals to be aware of and appreciate the agency they possess in influencing preferred ways of being (White 1991).

*Feminist disability theory* understands disability as a ubiquitous cultural system that classifies certain kinds of bodily differentiations. Like femaleness, disability is a culturally created narrative of the body (Garland-Thomson 2002). As with systems of gender and race, the ability or disability systems produce individuals through the marking and differentiating of bodies. This ideological comparison influences the formation of culture and legitimises the unequal distribution of power, status and resources within a biased social context (Garland-Thomson 2002). People with disabilities, as with women’s bodies, are subjected to what Foucault (1979) described as ‘discipline’, where systems of race, sexuality, ethnicity, gender, ability and class all place great amounts of social pressure to normalise, regulate and shape the subjects’ bodies. There is a strong push towards fixing and regulating differentiated bodies, often at the expense of producing a more accessible social context, or improving the provision of support to people with disabilities (Garland-Thomson 2002).

Power, according to Foucault (2000, as cited in Reeves 2002), is brought about in the body and is created in every human relationship; it does not reside with one individual but permeates throughout. Power and knowledge are intrinsically linked and one cannot exist without the other. Knowledge is what allows individuals to become subjects, as individuals use different bodies of knowledge as points of reference in understanding themselves and others. Disciplinary power categorises individuals, and subjects them to continuous forms of surveillance. This involves the creation of rules that allows for the monitoring of the body to ensure that it is useful (Reeves 2002). Reeves (2002) cited the following example: bodies of people of disabilities are under constant surveillance by medical practitioners who attempt to identify any form of defect to categorise that individual as a patient (the body has become an object of power or knowledge). Improvements in medical technology have allowed greater efficiency in the manner in which individuals classify and document the body. The visibility of an impairment allows any observer access to privileged information and thus power about their body. This power is influenced by assumptions and prejudices around disability and can prevent an individual from participating in society. Consequently, those that are subjected to the constant power of the gaze develop an awareness of their impairment and begin to engage in self-policing in an attempt to appear acceptable and ‘normal’. These tensions (multiple experiences [White 2001]), which are experienced by people with disability, provide moments of possibility in which to examine, re-create and expand their personal and relational identity (Foucault 1979).

The ontological and epistemological underpinnings of the above frameworks provide a structure in which to engage with the lived experiences of SWD.

## Aims and objectives

In this study, we aimed to explore the narratives around disability within an HEI. The study made specific inquiry into the experiences of SWD and key players who influence this experience, namely, lecturers and the institutions’ DU staff members. This bears significance for practice-level planning and policy-making in the area of disability.

## Study design

A qualitative research design was used as the researchers aimed to explore the personal, in-depth meanings and understandings of disability as well as the contextual factors that shape those meanings. The common-sense understandings of reality are important in qualitative research, as these contain the meanings that individuals use when they interact with others (Neuman 2006). It is idiographic and inductive in nature.

### Setting

The study was conducted with 12 SWD, five lecturers who teach SWD and seven DU staff members at the university. Participants who voluntarily agreed to participate in the study made arrangements with the researchers to meet for an interview at an agreed time on the institution’s premises. Interviews were approximately 1 h in duration and took place over a period of 2 weeks after the July mid-semester break in 2014.

### Study population and sampling strategy

Data were collected from three sources: SWD, lecturers who teach SWD and DU staff. As the research required a very specific sample of the tertiary population, purposive sampling was used. The researchers ensured that the sample selected included characteristics of the population with regard to race, gender and culture. Biographical questionnaires and semi-structured interviews were used in the collection of data.

Twenty-four participants were interviewed:

- Twelve SWD between the ages of 19 and 56 of which seven were male and five were female.
- Seven members of staff from the institutions’ DU were interviewed. Their ages ranged between 25 and 42, of which four were male and three were female.
- Five lecturers from within the school of applied human sciences were interviewed, of which three were male and two were female.

### Data collection

Once permission and ethical approval to conduct the study was granted, individuals who met the sample criteria were invited to participate. This was carried out through posting of notices around the institution about the study as well as approaching students, staff and lecturers on the campus. Those who voluntarily agreed to partake in the study made arrangements with the researcher to meet up for an interview on the institutions’ premises. Participants signed an informed consent form, outlining what the study involved. All participants were briefed about the study, their roles, matters of confidentiality and that their participation was entirely voluntary. With the permission of the participants in the study, interviews were audio-recorded. Only three participants declined audio recording and in those instances, the researcher requested permission to take down notes that were then transcribed immediately to maintain accuracy.

### Data analysis

The researchers transcribed the recordings of each interview verbatim, analysing the data using thematic analysis, as suggested by Braun and Clark (2006). The research analysis was located within a social constructionist epistemology (identifying patterns and themes as socially constructed), to facilitate an understanding of how the participants make sense of their disability, and how these are influenced by different socio-cultural contexts and conditions. The researchers used an inductive approach to thematic analysis.

### Ethical consideration

The researchers requested permission to conduct the study from the Higher Degrees Committee of the Faculty of Humanities, Development and Social Sciences at the University of KwaZulu-Natal (UKZN) as well as sought ethical approval from the Human Social Science Ethics Committee of UKZN (reference number: HSS/1349/013M). Furthermore, the researchers requested permission to conduct the study from the DU at UKZN. Once permission was granted, the researchers invited participants who met the sample criteria. Individuals who voluntarily agreed to partake in the study were asked to sign an informed consent form, outlining what the study involved. The researchers ensured that all information obtained during the study remained confidential and was only seen by the researchers, and that it would be kept in the Discipline of Psychology for a period of 5 years. The identity of all participants in the study was protected and under no circumstances was any identifying information mentioned. Participants were constantly reminded throughout the study that their participation was entirely voluntary, that they could withdraw from the study at any time and they would not experience any negative consequences for doing so.

In collecting the data, the researchers ensured that if at any stage, the participants experienced any negative consequences from the interview process, they would refer them to a registered psychologist to be debriefed and if required to receive counselling. Furthermore, the findings of the study were made available to the DU for the support unit to draw on the learnings to inform the support they provide to SWDs.

## Results

The findings suggest that in spite of the facilitating and positive representations of disability present in the institution, the dominant representation of disability was perceived as challenging, and as a result, disempowering. Students with disabilities were found to adapt, and consequently, modify their behaviour by disassociating from their disability in order to fit in. There is a strong emphasis on students having to adapt in a tertiary context. Through normalisation mechanisms of the ‘gaze’, through the engagement with people without disabilities and through the language used when speaking about SWD, these understandings are perpetuated and internalised.

As a main finding, ambivalence between needing to associate with being disabled and simultaneously disassociating with being disabled emerged from the data. In understanding the results, the researchers grouped the findings into three sub-themes:

- representations of disability
- methods of normalising disability
- the management of disability.

## Discussion of key findings

### Representations of disability

A clear disempowering representation of disability emerged from the data, one where varied embodiment or being different was seen as inferior. There was an ‘us and them’ tension that was present predominantly between SWDs and students without disabilities. This tension was also present in the interface and interaction between service providers (DU staff and lecturers) and SWD:

‘… [*W*]ith disability yeah, knowing the fact that there are some sort of stuff that you cannot do because you are one and two, somehow it automatically side-lines you, you know, there are some things that I for one as a student with disability I cannot do whereas another student can do ….’ (Participant 13, 23 years old, Male)

‘… I feel that there is a certain type of stigma around people with disabilities at the university, other people try to be helpful, you, when a person is with a disability we have the right to skip queues and stuff and you can feel that there is tension and people feel like this is unfair because we’ve been standing here for hours and stuff like that.’ (Participant 14, 21 years old, Female)

In the above excerpts, SWD 1 views himself as different, and therefore, he feels different and excluded. Student with disabilities 2 describes a tension that is present between SWDs and students without disabilities when SWD get preferences. It appears as if SWDs experience resentment from the students without disabilities when they utilise processes or facilities that assist them. A feminist disability framework highlights that people’s understandings of disability are formed through marking those who appear different (SWD) in comparison to the culturally accepted norm (the students without disabilities). The above excerpts both illustrate this process of marking, through making SWD feel different, their inability to do certain things that consequently make them feel excluded are highlighted as seen with SWD 1 and through the tensions that are created between students with or without disabilities as experienced by SWD 2. Many SWD in the study described how the students without disabilities are sympathetic towards them, and it is interpreted as if they are people who are less or are incapable of achieving things in the same manner as the students without disabilities; the following excerpts highlight these feelings:

‘Uh I think students generally pity students with disabilities like it’s a oh shame type of attitude and I think that needs to change yeah and they need to understand that there might be something physically wrong with us but we have the same mental capacity as them.’ (Participant 14, 21 years old, Female)

‘Students also have this sympathy, they feel sorry for SWD, people need to understand that we are disabled but it’s not like we can’t do things.’ (Participant 23, 22 years old, Male)

Individuals are able to exercise power by drawing from discourse. These understandings allow peoples’ behaviours to be represented in a particular way and highlight what is acceptable and unacceptable within a specific context (Burr 2003). Student with disabilities 2’s understanding of his disability as something ‘wrong’ indicates this view that his embodiment is less, it does not fit into what is considered as acceptable. Thus, when individuals represent or define something in a certain way, they are creating a form of knowledge that brings a form of power (Burr 2003). Both SWD in the above excerpts highlight feelings of frustration and agency when describing how they are just as capable as the students without disabilities and feel that they are not treated as such. A feminist disability framework would illustrate that the use of these categories in understanding and describing SWD can place them at a disadvantage through devaluing their bodies because they are non-conforming to culturally held standards in the institutional context (Garland-Thomson 2002). The people with disabilities are not only de-valued for their bodies (Hannaford 1985, as cited in Wendell 1989), but they are also reminders to the able-bodied of what they are trying to avoid, ignore or forget (Lessing, J., 1981, Denial and disability. *off our backs* 11(5):21.).

Disability systems work to validate and sustain certain privileged categories such as normal, fit and competent, which all create cultural power to those who claim to have that particular status and who live in these positions (Garland-Thomson 2002). The following interview where a male SWD describes an incident with a female friend of his without disability illustrates how this is played out:

‘I had a friend I was really close to who was female and I think that I don’t know we had like a weird relationship because we weren’t dating but at the same time we liked each other so we were always acting as if we were dating. But we always say, like no, my friends and what not and we call each other husband and wife. So one day, she came towards me, she was sitting with her friends and one of them was a guy, and she came towards me and she was like hugging and like, oh, this is my husband and what not – and the guy looked at the girl and said oh are they really dating? And the friend knew we weren’t really dating, but she was like – yeah they dating why? And he was like, oh does he have lots of money or something? So there was that idea that disabled people, disabled guys would only get girls, if they have cash and that attitude.’ (Participant 22, 22 years old, Male)

As the above excerpt illustrates, for the student without disability, the idea of a SWD being able to have a relationship with a student without disability did not fit into his categories of ‘normal’. The male SWD was understood as less, not being fit to date an able-bodied female. Therefore, there had to be an alternative reason for the existence of their relationship such as the SWD having wealth. Furthermore, this dynamic plays out between SWD 10 and his female friend without disability as well, he describes:

‘[*W*]e had like a weird relationship because we weren’t dating, but at the same time, we liked each other, so we were always acting as if we dating, but we always say like no my friends, and what not.’ (Participant 22, 22 years old, Male)

The idea of having a relationship was not considered the cultural norm and was therefore regulated within a public space.

Within a feminist disability framework, understandings such as these control differentiation and highlight hiddennorms of which bodies of people with disability arenot part of (Garland-Thomson 2002). Furthermore, these understandings perpetuate the characterisation of the people with disabilities as inadequate, redundant or restrained (Garland-Thomson 2002).

Students with disabilities are thus marked through systems such as these, and attempts are geared towards normalising or eliminating the differentiation through a number of cross-cultural actions (Garland-Thomson 2002).

However, there are alternative representations and discourses of disability present within the tertiary institution that challenge the view that SWD are less capable than the students without disabilities. This can be seen in the following excerpts:

‘… [*S*]o do not look at me and say oh you have big eyes, how does your body look, I do not see anything physical about you, do not do that, do not dictate as to what my disability could be and what it is, just treat me as a student.’ (Participant 16, 21 years old, Female)

‘They might have certain difficulties you know, they might have certain impairments, they might not be able to do certain things, they might not be able to walk with two legs like most people, they might have a skin condition or whatever it is you know, they might be different, but a lot of those people besides the fact that they sometimes can’t do certain things, they are unable to do certain things, they are human beings like everybody else.’ (Participant 6, 31 years old, Male)

A feminist disability framework would understand the above excerpts as more facilitating representations of disability, and these counter-narratives allow for ‘resymbolisation’ where opportunities are created to shape and retell culturally held beliefs about SWD and by doing so, influence their experience (Garland-Thomson 2002). This can further minimise the identification of SWD in terms of discriminatory and oppressive attitudes towards people with disabilities (Garland-Thomson 2002). A social constructionist view understands that personal narratives allow people to take on conceptions and ways of being that may be more facilitating (Garland-Thomson 1998) and may be more in line with their personal understandings. Highlighting this process allows individuals to be aware of and appreciate the agency they possess in influencing preferred ways of being (White 1991) as SDW2 describes, ‘just treat me as a student’. Thus, the students and staff members above have created more facilitating ways of understanding disability; in spite of SWD being different, they are no less than the students without disabilities and should be treated as such.

Within a Foucauldian perspective, power and resistance are seen as mutually related. The power inherent in one discourse is only apparent from the inherent resistance in another (Burr 2003). Thus, the above excerpts highlight Foucault’s notion of ‘discursive resistance’. This involves the emergence of multiple subject positions as alternatives to the dominant discourse (Caldwell 2007). Foucault understands discursive resistance as a positive productive force, rather than simply a negative counter reaction (Caldwell 2007). Discursive resistance is effectively a volitional act of refusal (Caldwell 2007). This is clearly illustrated in SWD 4’s quote; ‘do not dictate as to what my disability could be and what it is’. Discursive resistance allows those ‘subjects’ of power (SWD) to act otherwise and reject their confinement within predetermined discourses of power or knowledge (Caldwell 2007).

### Methods of normalising disability

The researchers found that SWD are subjected to what Foucault (1979, as cited in Garland-Thomson 2002) described as ‘discipline’, where systems of race, sexuality, ethnicity, gender, ability and class, all place great amounts of social pressure to normalise, regulate and shape subjects’ bodies (Garland-Thomson 2002). According to Foucault (1977, as cited in Reeves 2002), disciplinary power categorises individuals and subjects them to continuous forms of surveillance. It involves the creation of rules of normalisation that allows for the monitoring of the body to ensure that it is useful (Reeves 2002). For example, many SWD talk about how they are looked at as abnormal:

‘… [*I*]t’s all about you know what I’m saying about perception, they view you in a certain way if you’re a disabled person they look at you differently you not supposed to be that, so that’s how people view us disabled students I think.’ (Participant 21, 21 years old, Male)

‘So when you are disabled you I still have to start like, what kinds of people are meeting there, like so it’s a new environment, new people and we have to always, we always like stared at, people like some, the first time they see you, they stare, so all those experiences we live with them every day but you get used to it.’ (Participant 18, 21 years old, Female)

The SWD in the above excerpts were subject to what Foucault (1980) described as the power of the gaze, which occurs in their everyday social interactions (Reeves 2002). The visibility of an impairment of an SWD allows any observer access to privileged information and thus power over their body. This power of the gaze is influenced by assumptions and prejudices around disability and can exclude the people with disabilities from participating fully in society (Reeves 2002):

‘[*S*]ocially yeah well it’s very hard to make friends and communicate because I felt intimidated by, say they might judge, be judgemental, I’ll be judged because of my disability, so it’s hard for me to make any friends or you know, interact.’ (Participant 21, 21 years old, Male)

SWD 9 describes how difficult it is to socialise because of the fear of being judged as can be seen above: ‘I felt intimidated by say they might judge, be judgemental’. Further, an SWD who has a hidden impairment (such as a mental disability) is subjected less to the power of the gaze, but constantly fears being ‘discovered’ (Thomas 1999, as cited in Reeves 2002). These individuals might, however, still be subject to the gaze from others when utilising facilities for the people with disabilities (Reeves 2002). For example, a student with a psychological illness in the current study describes how her disability was ‘discovered’ and how others obtained access to privileged information about her body:

‘I decided to use my skip queue letter because the line now I can pass people then whenever I stand in the queue or whatever I do not feel like talking to people I do not feel like seeing people I don’t like being around people so I found myself being with people, and I was in a bad space so I decided to take out the skip queue letter for myself and I went and stood in the line towards the side to the third table, I went there and I showed the lady in front that I had this letter and then I stood. The person finished from the desk and then I proceeded forward; I don’t even remember what she said, but hurt me in such a way that I just broke down there and now it was seen and everything and yeah’. (Participant 21, 21 years old, Male)

A further method of normalising is through the language that is used when speaking about SWD. A social constructionist framework understands that it is through language that understandings of disability are constantly being constructed and perpetuated in the society (Burr 1995; Durrheim 1997). Through the language people use in their everyday interactions with one another, they actively produce forms of knowledge around disability (Burr 2003). Students with disabilities are spoken about by lecturers and DU staff in the current study in normalising ways, they are spoken about in terms of how they have ‘improved’, how ‘normal’ they are or how they ‘adjust’; the following excerpts illustrate this:

‘It’s like they adjust to their disability and they actually they do well even what’s this disability called, I forgot, but you know their speech actually even improves because I guess they interact with so many people when they here, that they actually, you know they improve, so I think that’s been a, that’s been a success for me to actually see people grow in that way.’ (Participant 10, 26 years old, Female)

‘… [*Q*]uite a lot of them don’t even, you know, being disabled is not even you know, they don’t even, I don’t know whether they actually think about it – I can’t obviously speak for them but it just doesn’t affect them when you talk to them and how they carry on with their lives or maybe that, you know, but they come out as people who are not disabled.’ (Participant 6, 31 years old, Male)

‘… [*T*]hey really, they do not let their disability get the better of them.’ (Participant 2, 26 years old, Female)

‘[*T*]o see the students are going about, the disabled students are going about with their student lives on their wheelchairs, electronically, walking around with the walking stick, so there is that sense of normality which I think sort of for me is a positive thing.’ (Participant 5, 48 years old, Male)

As these excerpts illustrate, language thus has a normalising function. The manner in which disability is spoken about in a tertiary context is one where a greater emphasis is placed on the student having to fit into the environment. This emphasis on students having to ‘fit in’ minimises the tolerance for human difference within a university context, as this emphasised the understanding of disability in bodies as flawed, rather than the need for social systems to be more responsive and in need of review (Garland-Thomson 2002).

Foucault describes these normalising methods as ‘dividing practices’ – modes of manipulation that make up a scientific discourse with practices of social exclusion and segregation to classify, distribute and manipulate subjects (Tremain 2001). Through these practices, SWD (subjects) become objectivised, such as healthy or sick, able bodied or disabled (Tremain 2001). Morris (1991)highlighted that behind these techniques lie prejudices around the value of lives of individuals with disabilities. Individuals with disability are often devalued through this form of power and are made to feel a sense of unworthiness and rejection (Reeves 2002). As a consequence, SWD who are subjected to these ‘dividing practices’ develop an awareness of their impairment and begin to engage in self-policing in an attempt to appear acceptable and ‘normal’ (Reeves 2002).

### The management of disability

Many SWD in the current study described disassociating with being disabled and acting in ways that limit being treated differently so as to fit in; the following excerpts illustrate this:

‘I’m one that doesn’t like wearing my glasses all the time because I think it attracts unnecessary attention, so I have to walk around half blind at times and there are certain things that I would see and there are certain things that I wouldn’t see and there are certain things that I would choose to see and would want to really see and there’re certain things that I’m like, well, I don’t really need to see that. And unfortunately it goes with, to a certain extent it goes with a choice, and um … and for someone, who can see properly, you don’t choose to see things, right? So for me, there are certain things that I would choose to see.’ (Participant 16, 21 years, Female)

‘… [*U*]h during registration you don’t hold, you don’t stand in the queue, if you stand in the queue it’s because you like, like I myself, I for one, stand in the queue because I don’t want to be treated differently.’ (Participant 13, 23 years, Male)

‘[*T*]he problem about myself is that I don’t, like I don’t, I know that I am disabled, I’m using crutches and so forth, but I try to live my life as how a non-disabled student lives his or her life.’ (Participant 15, 22 years old, Male)

‘… [*P*]ersonally I keep away like I’m to myself and I don’t really sit out you know – because of this fear of being judged as the only person with a disability.’ (Participant 21, 21 years old, Male)

Within a Foucauldian perspective, the above actions are forms of self-surveillance, and all methods of normalisation work together to ensure that SWD (subjects) have a self-assessing, self-monitoring and reflexive relation to themselves (Hook 2007). Students with disabilities start to live as if they are under constant surveillance and become the sole controllers of their regulation (Hook 2007). As illustrated above, even though not wearing her glasses will further debilitate SWD 4, she would rather not wear them to fit in.

The dynamic of students associating with their disability but simultaneously disassociating with their disability to fit in is highlighted as well. Students with disabilities thus internalise current understandings of disability within this context and perpetuate it by sustaining the *status quo* (Hook 2007). For example, the SWD below describe how they need to take responsibility of managing their disability:

‘[*I*]t boils down to being disciplined uh, you act professionally even though you maybe you may differ in which other way, some of the other things that people do but you try and like supress your emotions, you compromise, something’s are hard to solve, you have to compromise

… it’s the individual that has the power to do what he or she wants to do, it’s not, it’s not a collective thing whereby you can wait for somebody to do something for you, you should do it yourself you, if you have a grievance you should take up the relevant department or generally people who have authority to solve such things.’ (Participant 15, 22 years old, Male)

‘… [*I*]t just depends on the individual student as I’ve said whether you make the effort, whether you get yourself out there or you choose to you choose to be very passive and you choose to let people come to you and whatever the case instead of going out there and actually getting these things yourself yeah.’ (Participant 16, 21 years old, Female)

As the excerpts above illustrate, the representation of disability places greater emphasis on the student, having to adapt and fit into the tertiary environment as being internalised and perpetuated. Knowledge around what is normal and what is culturally acceptable in terms of the body are the different forms of knowledge and understanding that are used as points of reference in understanding themselves and others, and by doing so they become objects of power or knowledge (Foucault 1977, as cited in Reeves 2002). As Tremain (2001) described, subjects are productive because the outcome of surveillance is to make the individual an object of knowledge that brings about a particular truth about disability. Furthermore, subjects are productive because the truth that is taken on improves its utility, making it more compliant, calculable and comprehensible (Tremain 2001), as SWD 4 describes, ‘it just depends on the individual student, as I’ve said, whether you make the effort’.

## Strengths and limitations

The current study used a qualitative research design, purposive sampling methods and involved in-depth interviews with a specific sample of participants within a specific context (lectures, the DU staff and SWD within an HEI). Using this approach enabled researchers to conduct an in-depth exploration of this phenomenon by examining how these individuals personally describe and articulate how they make sense of disability and the related issues in this context. The researchers did cross-check understandings during the interview process; however, they did not carry out follow-up interviews that could have provided participants with the opportunity to verify the data analysis and ensure accuracy (Given 2008). Furthermore, the current study included key stakeholders who had a direct impact on the experience of SWDs and did not include the voices of the able-bodied students who could have provided a further understanding into the experiences of disability within an HEI.

## Recommendations

The creation of positive representations of disability was highlighted in the study as important to all participants. This was highlighted even though there was an acknowledgement of the inherent infrastructural and financial constraints. Acknowledging this, students without disabilities and key stakeholders within a tertiary context should work on creating positive representations of disability at all levels of the institution, including the manner in which SWD are spoken about publicly, through engagement with SWD and HEI policy.

Developing and running awareness and education campaigns around disability as well as the role of the DU is vitally important in the creation of these positive representations and for challenging dominant representations.

These campaigns need not be resource-intensive but should occur regularly and be an inherent part of the DU’s mandate to educate the tertiary community on disability issues. It further creates greater visibility of the DU and its functions. More importantly, key stakeholders should be a part of this process in enabling the right issues to be addressed. Specific education for DU staff and lecturers is important as well, as these individuals engage directly with SWD.

Communicating with key stakeholders was highlighted as another concern in the study. When developing new initiatives for the DU or for the institution regarding disability, there needs to be a consultative process with SWD, the DU staff and lecturers. Here again, this need not be an expensive process and can be a simple informal conversation on what the needs of SWD, the DU staff and lecturers are. This is important as these are the stakeholders who are engaging with SWD on a daily basis and will probably have the best suggestions on how to address the issues concerning the SWD.

Greater support is required from the institution in providing accessibility to SWD, especially with regard to providing basic services. Any institution that aims to serve people with disability has to support this goal through the services they provide. Doing so sends out a strong message to the entire tertiary community and society at large that SWD are valued members of the institution, and it helps to create positive understandings of disability from the very top of the institution to the bottom of the hierarchy.

Finally, ensuring integration at all levels of the institution is important in challenging dominant narratives of disability. This involves ensuring that SWD are consulted or represented on all committees or forums that have an impact on their tertiary experience, such as having a representative on the Student Representative Council, any sporting councils or when improving or constructing new infrastructure within the institution.

## Conclusion

The dynamics of SWD disassociating with their disability whilst simultaneously needing to identify with it appears to occur within a system of normalisation. Although there are more facilitating and positive representations of disability in the institution, the dominant representation of disability within this context is one that is disempowering and understands different embodiment, as less. There is a strong emphasis on students having to adapt in a tertiary context. Through normalisation mechanisms of the ‘gaze’, through the engagement with the students without disabilities and through the language used when speaking about SWD, these understandings are perpetuated and internalised. Consequently, many SWD modify their behaviour and act in ways to fit in and disassociate with being disabled. Furthermore, many believe that they have to take ownership for their disability and manage it. The study highlights the need for creating spaces and engagement within an HEI that celebrates and creates positive representations of disability. Doing so will create opportunities to challenge the fundamental make-up of the current disempowering understandings of disability, as well as the possibility of changing these representations. Greater illustrations of positive representations can improve the manner in which access is provided to SWDs and can create understandings of the disabilities of the SWD, who have a right to equal access to infrastructure, resources and processes, enabling them to learn on an equal footing as their able-bodied counterparts.
